# A Rediscovery of *Arachnopeziza fitzpatrickii* Korf (Arachnopezizaceae, Leotiomycetes)

**DOI:** 10.3390/life16101665

**Published:** 2026-10-04

**Authors:** James K. Mitchell, Donald H. Pfister, Luis Quijada

**Affiliations:** 1 Department of Organismic and Evolutionary Biology, Farlow Herbarium, Harvard University Herbaria, Harvard University, Cambridge, MA 02138, USA; jmitchell@g.harvard.edu (J.K.M.); dpfister@oeb.harvard.edu (D.H.P.); 2 Departamento de Botánica, Ecología y Fisiología Vegetal, Facultad de Farmacia, Universidad de La Laguna, Avenida Astrofísico Francisco Sánchez, s/n, Apartado 456, 38200 San Cristóbal de La Laguna, Tenerife, Canary Islands, Spain

**Keywords:** helotiales, New England, marginal hairs, rare fungi, taxonomy

## Abstract

In 2019, *Arachnopeziza fitzpatrickii*, a species previously known from only three collections, was collected in Massachusetts. The species is unusual in *Arachnopeziza* in that it consistently produces fascicles of hairs at the margin of the apothecia, a character that led Korf, when describing the species, to consider that it could belong to genus separate from *Arachnopeziza* This specimen of *A. fitzpatrickii* was examined morphologically in a living and dead state, and two gene regions (ITS and LSU) were sequenced for phylogenetic analyses. The results of our phylogenetic analyses indicate that the genus is polyphyletic. *Arachnopeziza fitzpatrickii* is recovered in *Arachnopeziza* sensu stricto though it occupies an isolated position in the genus distant from other species producing fascicles of hairs. Observations of our specimen are largely in agreement with the original and updated published descriptions of *A. fitzpatrickii*, with only a few features differing; these disagreements are likely attributable to the earlier descriptions being based on observations of dead material. Comparisons are also provided of all species in *Arachnopeziza* producing fasciculate hairs, whether marginal or elsewhere on the receptacle.

## 1. Introduction

On 4 May 2019, during the Friends of the Farlow’s annual Clara Cummings Walk at Harold Parker State Forest, JKM collected several old acorn caps bearing *Arachnopeziza* species. Most of the specimens of *Arachnopeziza* were identified as *Arachnopeziza aurelia*, a common spring species in New England. A specimen including smaller, white apothecia was also collected, and upon microscopic examination and consultation with LQ and DHP, these apothecia were identified as *Arachnopeziza fitzpatrickii*.

*Arachnopeziza fitzpatrickii* was first collected by Richard Korf on old wood in Ithaca, New York in May 1947, and he later described it in his 1952 monograph of tribe Arachnopezizeae [1]. It was the first species he ever described [2]. At the time, he noted that it was unusual in *Arachnopeziza* in having hairs in fascicles only at the margin (teeth-like), which he illustrated. He placed it in an unranked infrageneric taxon he called “*Arachnasterae*.” While recognizing its distinctive characters and noting that it could be separated from *Arachnopeziza* on the basis of these characters, he did not consider *A. fitzpatrickii* different enough to warrant placement of the species in a separate genus. The species was not collected again for years despite efforts to collect from the same log in the type locality before the area was destroyed in a gravel-digging operation [3]. It was not until 1975 that he saw it again, this time collected by his student, Martha Sherwood, on decorticated wood in Alleghany National Forest, Pennsylvania in October 1975. This specimen, unlike the scant type preserved at CUP, was plentiful, and duplicates were sent to FH, K, and NY in addition to being preserved at CUP. A specimen collected in Nagano Prefecture, Japan in April 1993 was also identified as *A. fitzpatrickii* and is preserved at TNS [4].

While Korf [1] described several species of *Arachnopeziza* as producing fasciculate excipular hairs to varying degrees, the regular presence of the fascicles of hairs only at the margin (teeth-like) was a character he considered unique to *A. fitzpatrickii*. Recently, however, Kosonen et al. [5] found and described a species with the same morphological feature: *A. ptilidiophila*. This species was found in Finland, growing on liverworts in the genus *Ptilidium* and on adjacent *Pinus* wood. This species was found in their phylogenetic analyses to belong to *Arachnopeziza* sensu stricto and was further recovered in a distinct bryicolous clade in *Arachnopeziza*.

The aim of the present work is to document the rare *A. fitzpatrickii* morphologically and molecularly. A description based for the first time on living material along with micrographs of the macro- and micromorphology will aid in the future recognition of this species. Its molecular characterization will also help in detecting it in environmental and other sequencing studies. Molecular analyses also allowed us to test Korf’s placement of the species in the genus *Arachnopeziza* and to determine whether it is closely related to other species with fasciculate hairs.

## 2. Materials and Methods

The specimen of *Arachnopeziza fitzpatrickii* was found on fallen acorn cupules of *Quercus rubra* from the previous autumn buried under fallen leaves under *Q. alba*. The tree was located at the western edge of a powerline cut; the forest behind it was mixed, being primarily composed of *Pinus strobus*, *Quercus rubra* and *Q. alba*. The collection is preserved in the Farlow Herbarium, part of the Harvard University Herbaria (FH).

Fresh fruitbodies were studied following Quijada [6]. Congo Red, potassium hydroxide (KOH), Melzer’s reagent, Lugol’s solution, cornmeal agar (CMA), and chloramphenicol were purchased from the Sociedad Micológica de Gran Canaria (Las Palmas de Gran Canaria, Spain). Apothecial hand sections were cut under a Leica EZ4 dissecting microscope (Leica Microsystems (Schweiz) AG, Heerbrugg, Switzerland) and mounted in tap water or 1% aqueous Congo Red to observe living cells. For observation of dead cells, material was pretreated with 10% aqueous KOH or mounted in Melzer’s reagent. Microscopic features were studied using a Motic B1 compound microscope (Motic China Group Co., Ltd., Xiamen, China). Microphotographs were taken with a USB Moticam 2500 camera (Motic China Group Co., Ltd., Xiamen, China) and processed with Motic Images Plus v. 2.0. Microscopic measurements were conducted following Baral [7], also using his abbreviations for reagents and distinguishing observations of living and dead cells using the symbols * (living cells) and † (dead cells). Other abbreviations used are CR = aqueous Congo Red, LUG = Lugol’s solution, MLZ = Melzer’s reagent, and VBs = refractive vacuolar bodies. Color codes used follow Anonymous [8]. A culture was prepared on CMA supplemented with chloramphenicol using ascospores discharged from living apothecia, as described by Karakehian et al. [9]. This culture was later air-dried and is included with the original specimen deposited in FH.

**Figure 1:**
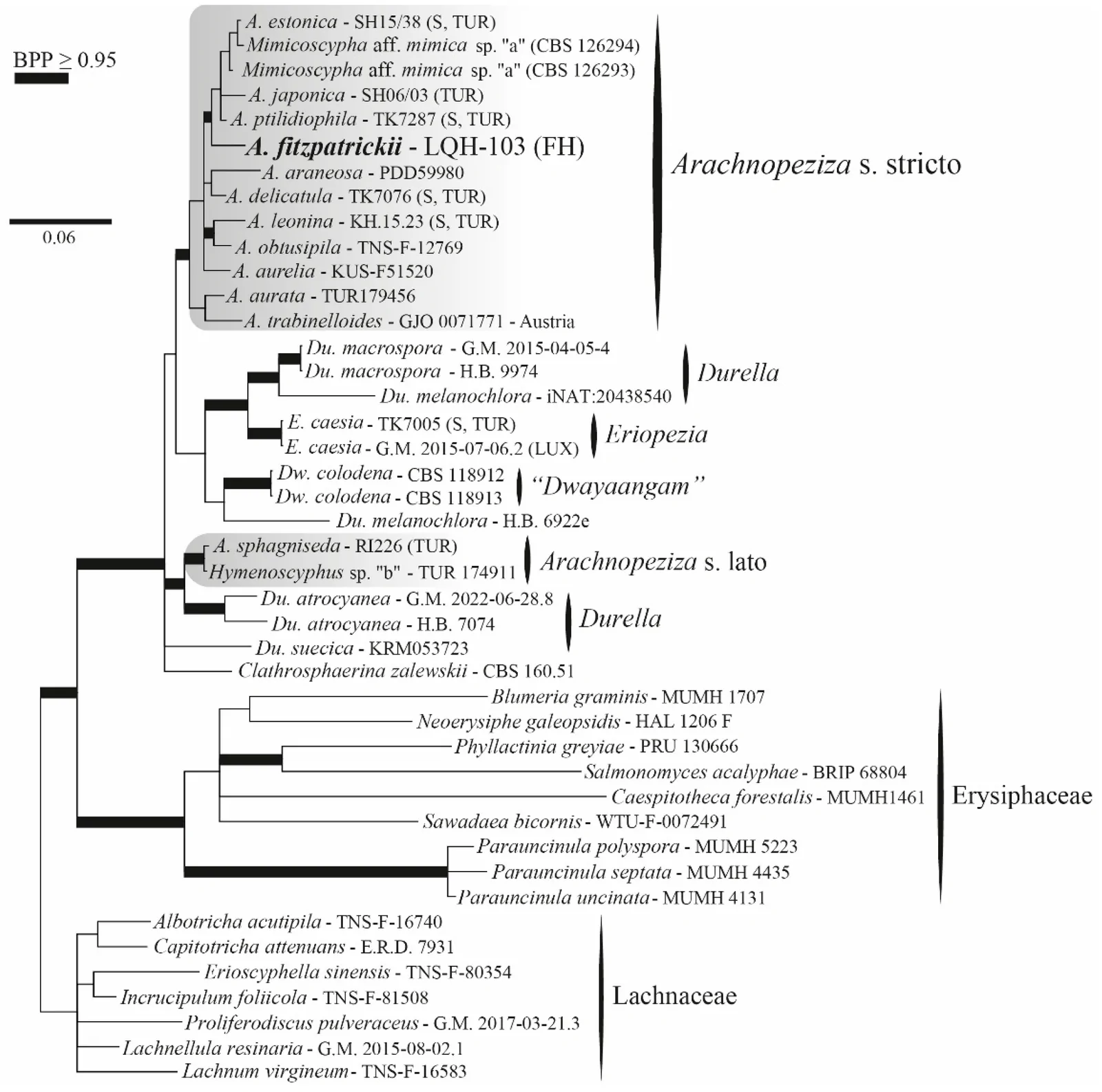
Phylogenetic relationships among *Arachnopeziza* species and representatives of related genera inferred from the concatenated ITS + LSU dataset using Bayesian inference. Thick branches indicate Bayesian posterior probabilities of 0.95 or higher. Gray shading identifies clades containing species previously placed in *Arachnopeziza*.

**Figure 2:**
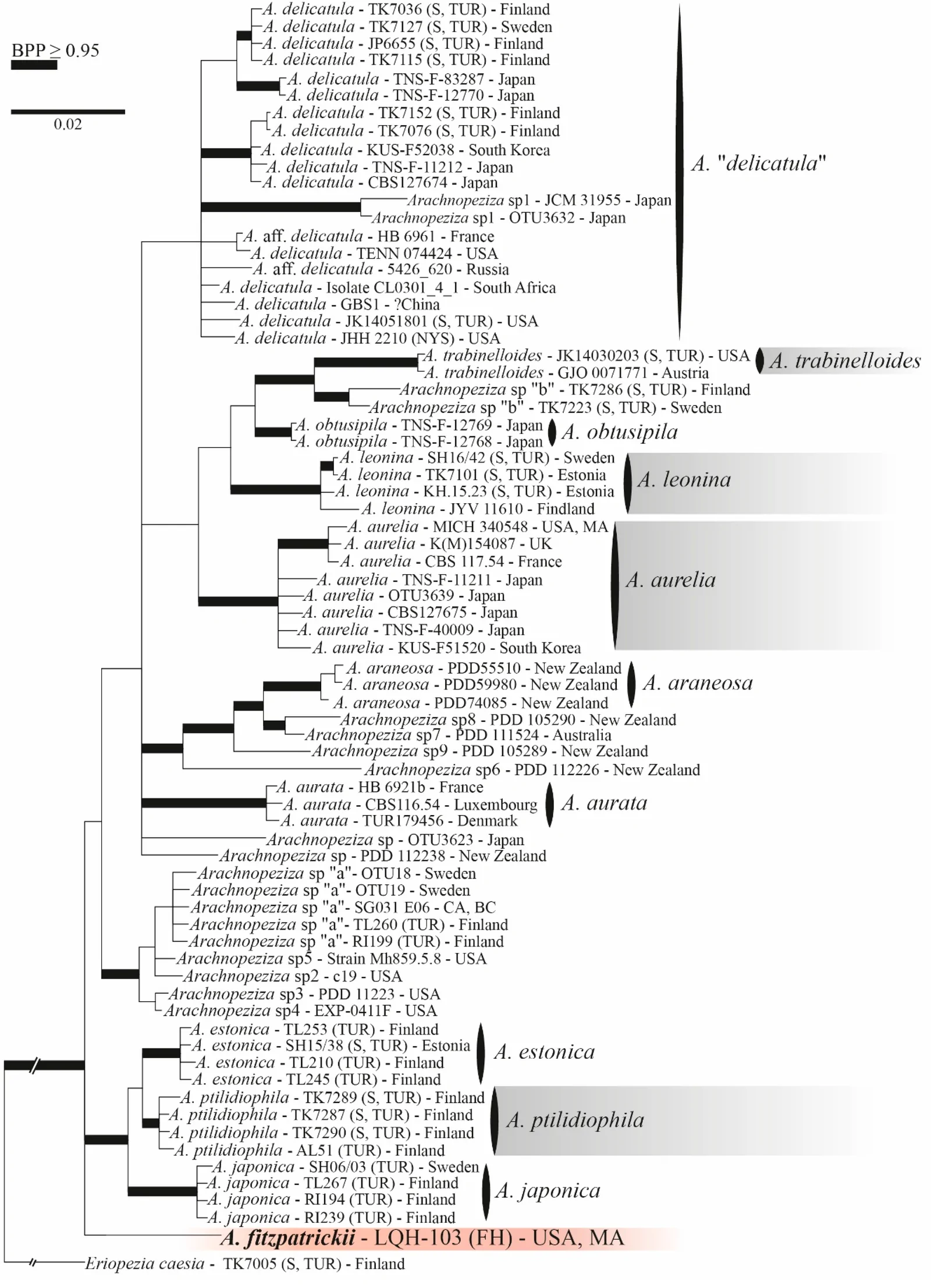
Phylogenetic relationships among *Arachnopeziza* species and strains inferred from the concatenated ITS + LSU dataset using Bayesian inference. Thick branches indicate Bayesian posterior probabilities of 0.95 or higher. Taxon names highlighted in gray indicate taxa producing hairs arranged in fascicles.

DNA extraction, PCR reactions, sequencing, and sequence assembly were performed as described in Mitchell et al. [10]. One fresh apothecium was used for DNA extraction. The ITS (internal transcribed spacer) and LSU (large subunit ribosomal ribonucleic acid) regions were amplified with the ITS1F/LR3 [11] and LR0R/LR5 [12,13] primer pairs, respectively, and sequenced. Sequence data for 104 terminals were included in the two phylogenetic analyses: 73 terminals assigned to *Arachnopeziza* and 31 terminals representing other genera. The ITS and LSU sequences of *A. fitzpatrickii* were newly generated in this study, whereas sequence data for the remaining terminals were obtained from GenBank (Table 1). Two rDNA gene regions (ITS + LSU) were used for the phylogenetic analyses. Separate ITS and LSU alignments were generated for each of the two phylogenetic analyses using MAFFT v. 7.017 [14]. The ITS region was aligned with the L-INS-i algorithm and the LSU region with the E-INS-i algorithm. After GBLOCKS filtering, the corresponding ITS and LSU alignments were concatenated separately for each analysis to produce the final matrices used for Bayesian inference. The final concatenated matrices comprised 43 terminals for the first analysis (Figure 1) and 73 terminals for the second analysis (Figure 2). The program GBLOCKS v. 0.91b [15] was used with the sequence type set to DNA to remove ambiguously aligned regions. The two datasets were filtered independently using dataset-specific parameters under the relaxed block-selection strategy [16], characterized by shorter final blocks, less stringent flanking-position requirements, and the retention of columns containing gaps in a limited proportion of sequences. For the first analysis (Figure 1), the minimum numbers of sequences required for conserved and flanking positions were 22 and 35 for ITS and 20 and 32 for LSU, respectively; for the second analysis (Figure 2), they were 39 and 40 for ITS and 24 and 24 for LSU. The maximum number of contiguous non-conserved positions was 4 for the first analysis and 10 for the second; both analyses used a minimum block length of 5 and allowed gaps in no more than half of the sequences. For the first analysis, GBLOCKS retained 363 of 758 ITS positions (47.9%; 395 removed) and 557 of 934 LSU positions (59.6%; 377 removed), yielding a 920-position concatenated matrix representing 54.4% of the initial 1692 positions. For the second analysis, GBLOCKS retained 432 of 530 ITS positions (81.5%; 98 removed) and 632 of 888 LSU positions (71.2%; 256 removed), yielding a 1064-position concatenated matrix representing 75.0% of the initial 1418 positions. The GTR + G+I nucleotide-substitution model was specified a priori for both phylogenetic analyses as a flexible, parameter-rich model, following the rationale of Abadi et al. [17]. In each analysis, the concatenated ITS + LSU matrix was treated as a single partition, with one set of substitution-model parameters estimated across all sites. Because no locus-specific partitions were defined, linking or unlinking parameters among partitions and specifying locus-specific rate multipliers were not applicable. This relatively simple partitioning scheme was chosen to avoid estimating separate parameter sets from a short matrix in which LSU was unavailable for many terminals, and because the primary aim was to assess the overall phylogenetic placement of *A. fitzpatrickii* rather than locus-specific evolutionary patterns. Bayesian inference (BI) was performed with MrBayes v. 3.2.6 [18] through the MrBayes plugin implemented in Geneious v. 6.1.7. Both analyses used the same MCMC protocol. Two independent runs, each comprising four chains (one cold and three heated; temperature parameter = 0.2), were configured for 4.6 million generations, with trees and model parameters sampled every 2400 generations. Rate heterogeneity was approximated using four discrete gamma categories. The first 250 sampled states from each run, corresponding to 600,000 generations, were discarded as burn-in. An unconstrained Gamma–Dirichlet prior [GammaDir(1.0, 0.1, 1.0, 1.0)] was applied to branch lengths, and an exponential prior with rate 10.0 was used for the gamma-shape parameter; all remaining priors were left at their MrBayes default values. Convergence was monitored in both analyses using the average standard deviation of split frequencies (ASDSF) between the two independent runs until values below 0.01 were reached, with values of approximately 0.009 attained in each analysis; this threshold was used as the convergence criterion. The first BI analysis tested whether *A. fitzpatrickii* was nested within *Arachnopeziza* and included 43 terminals: 27 ingroup terminals representing *Arachnopeziza* and related genera, together with nine Erysiphaceae and seven Lachnaceae terminals used as outgroups. The second BI analysis assessed phylogenetic relationships among *Arachnopeziza* species and included 73 terminals: 72 assigned to *Arachnopeziza* and one terminal of *Eriopezia caesia* used as the outgroup. In the first analysis, 37 terminals were represented by both ITS and LSU, five by ITS only, and one by LSU only. In the second analysis, 42 terminals were represented by both loci, 30 by ITS only, and one by LSU only. For terminals lacking one of the markers, the corresponding region of the concatenated matrix was filled with gap characters and treated as missing data in the Bayesian analysis. For more details about phylogenetic methods see Quijada et al. [6]. Phylogenetic trees were drawn with FigTree v. 1.4 and the artwork was prepared in Adobe Illustrator CS5. 

## 3. Results

The final 43-terminal ITS + LSU matrix used for the first phylogenetic analysis comprised 920 aligned positions. When gaps and ambiguous nucleotide codes were treated as missing, 330 sites were variable and 244 were parsimony-informative. The ITS region contained 168 variable and 136 parsimony-informative sites, whereas the LSU region contained 162 variable and 108 parsimony-informative sites. The final 73-terminal ITS + LSU matrix used for the second phylogenetic analysis comprised 1064 aligned positions. When gaps and ambiguous nucleotide codes were treated as missing, 191 sites were variable and 135 were parsimony-informative. The ITS region contained 138 variable and 104 parsimony-informative sites, whereas the LSU region contained 53 variable and 31 parsimony-informative sites. Clades with named genera and species are indicated in the BI phylogenetic analyses shown in Figure 1 and Figure 2, respectively. Our first phylogenetic analysis shows that the genus *Arachnopeziza* is polyphyletic (Figure 1). One clade contains all species (*sensu stricto*) but *A. sphagniseda* (*sensu lato*), which is not related to the type species of the genus, *A. aurata* (Figure 1). In the second analysis (Figure 2), all named species are monophyletic but *A. delicatula*. *Arachnopeziza fitzpatrickii* is not closely molecularly related to any species for which sequences are available.

**Figure 3:**
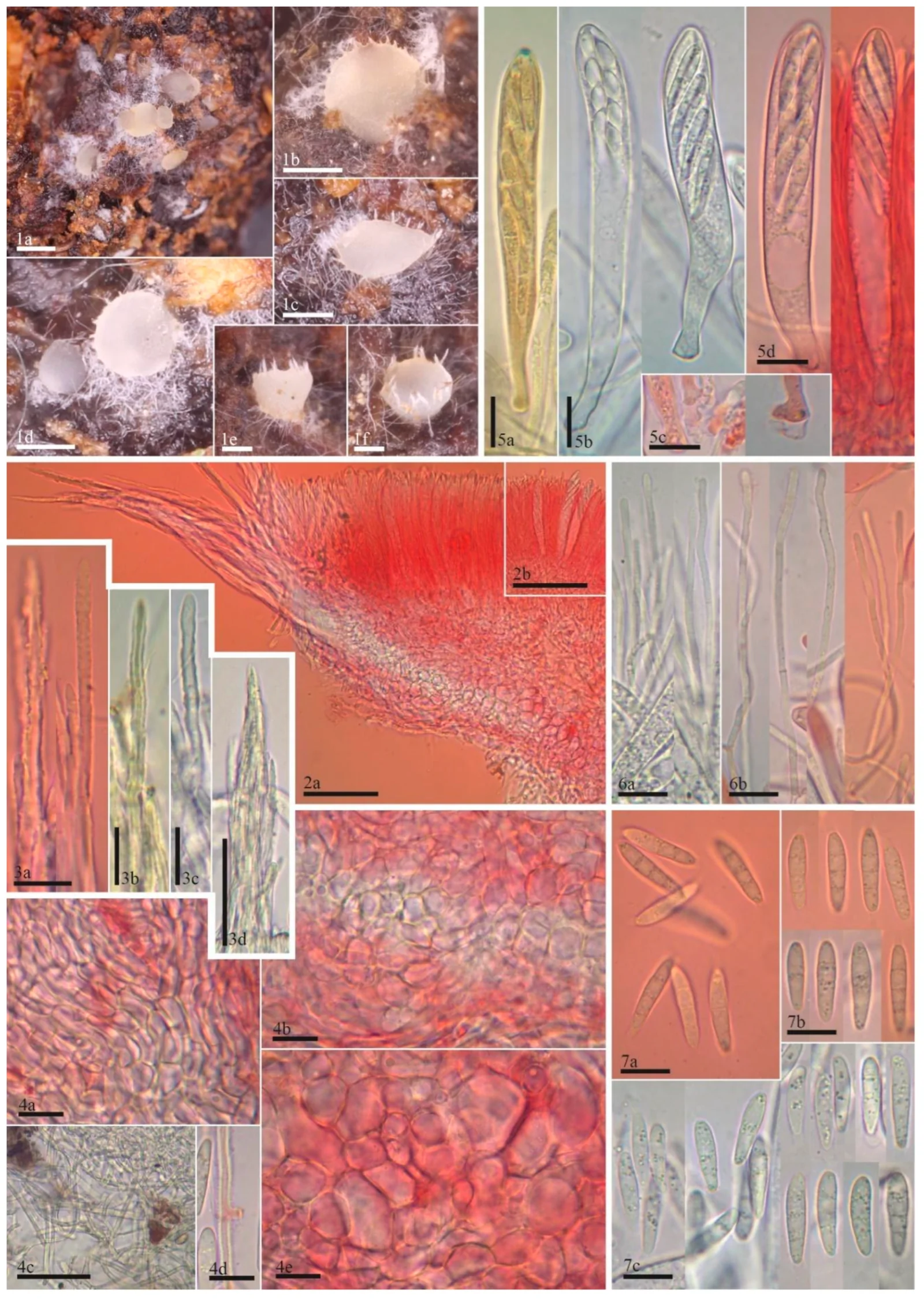
Detailed morphology of *Arachnopeziza fitzpatrickii*. (**1a**–**1f**). Fresh apothecia; (**2a**,**2b**). Transverse section showing details of ectal and medullary excipulum, hairs and hymenium; (**3a**–**3d**). Hairs, (**3a**). Living hairs in CR showing ornamentation, (**3c**,**3d**). Living hairs in water, (**3b**). Dead hairs in MLZ; (**4a**–**4c**). Excipular cells and subiculum, (**4a**). Ectal excipulum at upper flank and margin, (**4b**). Ectal excipulum at lower flank, (**4c**,**4d**). Subicular hyphae, (**4e**). Detail of ectal excipular cells at base of apothecium; (**5a**–**5d**). Asci, (**5a**). Dead asci with amyloid plug in MLZ, (**5b**,**5d**). Living asci in water and CR, (**5c**). Asci arising from croziers; (**6a**,**6b**). Living paraphyses in water and CR; (**7a**–**7c**). Living immature (aseptate) and mature ascospores (3-septate). Scales: 1 mm = (**1a**–**1f**); 50 µm = (**2a**,**2b**,**4c**); 10 µm = (**3a**–**3d**,**4a**,**4b**,**4d**,**4e**,**5a**–**5d**,**6a**,**6b**,**7a**–**7c**).

**Figure 4:**
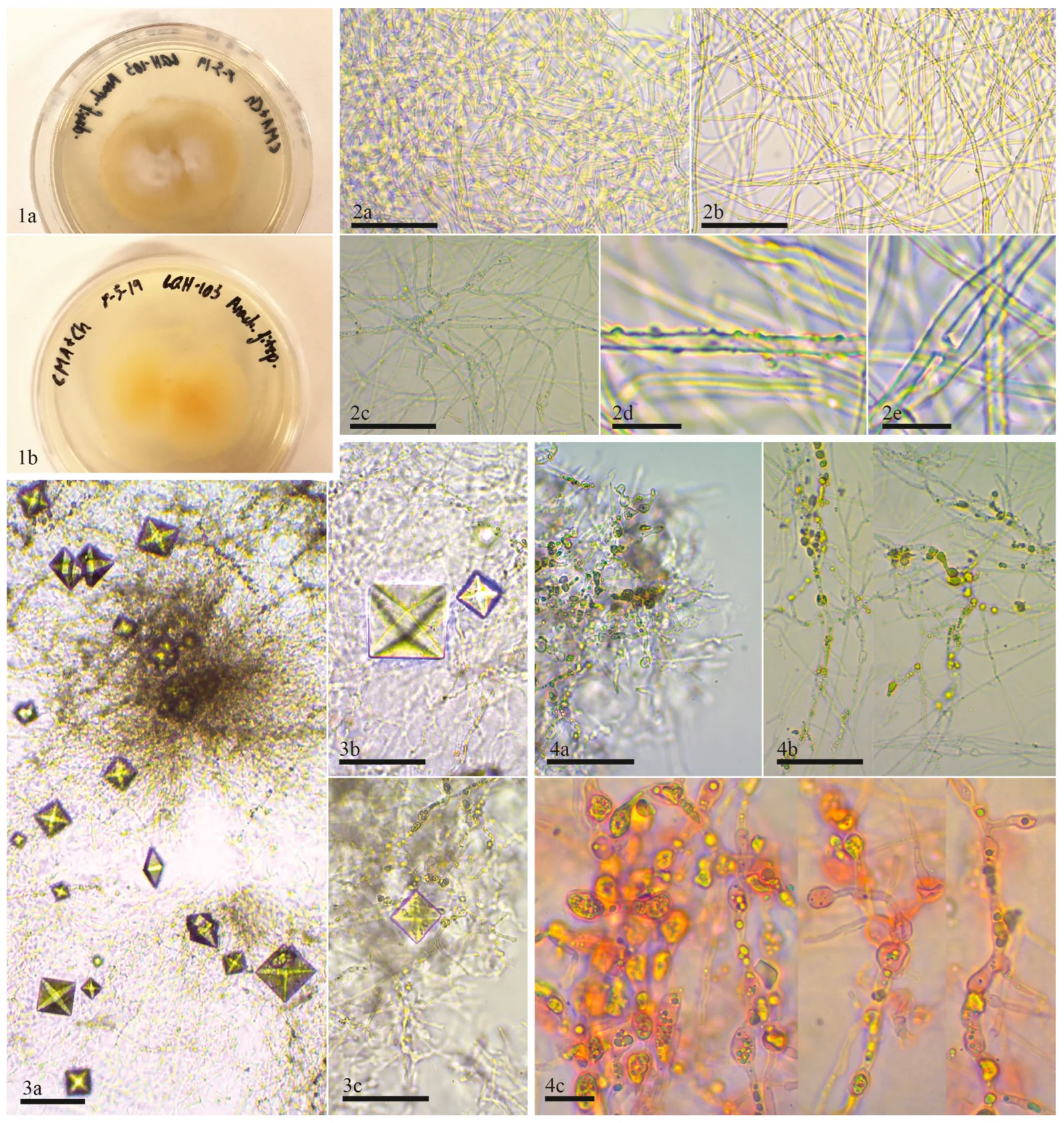
Culture of *Arachnopeziza fitzpatrickii*. (**1a**,**1b**) Macroimages of the mycelium growing on the Petri dish; (**2a**–**2e**). Hyphae in the center of the culture close to the margin; (**3a**–**3c**). Marginal hyphae with oxalate crystals; (**4a**–**4c**). Chlamydospore-like bodies at the margin of the culture. Scales: 100 µm = (**3a**); 50 µm = (**2a**–**2c**,**3b**,**3c**,**4a**,**4b**); 10 µm = (**2d**,**2e**,**4c**).

***Arachnopeziza fitzpatrickii* Korf, Lloydia 14(3): 168 (1952) [1951]; Figure 3 and Figure 4.**

**Apothecia** 0.6–1 mm diam., discoid-turbinate, up to 0.2–0.4 mm high, scattered to sub-gregarious, sessile; disc white (263.White) to pale yellow (89.pY), receptacle concolorous, margin with hairs formed in tooth-like fascicles, 8–16 fascicles up to 0.2 mm, subiculum white (263.White) sparse or abundant only close to the fruitbodies. **Hairs** cylindrical, slightly tapering toward the apical cells with a blunt apex, hyaline, (2) 3–4 (6)-septate, straight to slightly wavy, with sparse, hyaline amorphous grains, at margin *83–170 (206) × 1.5–2.5 µm; walls *† 0.4–0.5 (1) µm, slightly thickened and roughened, not changing in MLZ with or without KOH pretreatment, without guttules. **Asci** * (66.5) 76.5–85 (98.5) × * (8.5)10–11.5 (14.5) µm, † (59) 64.5–71.5 (76) × (7.5) 8–8.5 (9) µm; cylindric-clavate, 8-spored, spores 2-seriate, *pars sporifera* * 30–50 µm, apex obtuse to subacute, pore amyloid (*Calycina*-type) in MLZ and LUG with and without KOH pretreatment; arising from croziers. **Ascospores** * (14.5) 15.9–16.7 (18.6) × (2.8) 3.5–3.8 (4.5) µm, † (12.5) 13.6–14.7 (15.9) × (2.2)2.5–2.9 (3.3) µm; clavate to ellipsoid-clavate, straight or slightly curved, (1)3-septate, constricted at septa, hyaline, thin-walled, with sparse grayish green-yellow (105.gy.gY) VBs. **Paraphyses** cylindrical, uninflated, hyaline, 1–3-septate, not exceeding the asci; terminal cell * (15) 22.5–32.5 (46) × 1.5–2 µm, cell below * (13) 16–22.5 (25) × 1–2 µm; bifurcate at lower cells, thin-walled, without guttules. **Medullary excipulum** * 35–60 µm thick, hyaline, composed of *textura intricata*. **Ectal excipulum** * 35–59 µm thick at base and middle flanks and composed of *t. globulosa* to *t. angularis*, at margin and upper flank *t. angularis* to *t. prismatica*, * 20–47 µm thick; hyaline, without exudates, with sparse VBs (*0.5–1.5 µm diam.). Ectal cells * (10) 12–14 (19) × (6.5)7.5–8.5(10.5) µm at base to lower flank, * (7.5) 9.5–11 (15.5) × (3) 5–6 (8) µm at upper flank to margin, wall thickness * 0.5–1 µm. **Subicular hyphae** * 2–3 µm thick; straight or wavy, hyaline, walls slightly thick and ornamented with spines, sparsely septate.

**Cultural characters**: slow growing on CMA, mycelium first appressed and later submerged, cottony, margin even, center white (263.White), with aerial mycelium, extremes changing to light orange yellow (70.l.OY) without aerial mycelium. Hyphal cells close to the center of the culture shorter and wider, † 18–24 × 2.5–3 µm, wavy, more packed, branched and running in all directions; close to the margin cells growing radially, longer and thinner, † 37–46 × 1.5–2.5 µm, walls slightly thick and refractive, sometimes encrusted with deep yellow (88.d.Y) exudates (Figure 3(2d)). Margin of the colony with contorted and forming oxalate crystals (15–63 diam), cells intercalary, chlamydospore-like bodies, † 4.5–10 × 3.5–5.5(9.5) µm, with lipidic guttules. Without conidia.

**Specimens studied:** USA. Massachusetts: Essex County, Harold Parker State Forest, North Andover, westside of power line cut, southeast of Salem St., ca. 42°38′10″ N 71°02′50″ W 40 m asl, growing on acorn caps (*Quercus rubra*), buried under leaf litter under a *Q. alba* tree, edge of mixed forest with *Pinus strobus*, *Q. rubra*, *Q. alba*, etc., 4.V.2019, J.K. Mitchell & L. Quijada LQH-103 (FH!)

## 4. Discussion

Most morphological details from the original description of *A. fitzpatrickii* and our own observations agreed although we have found some morphological differences. Korf [1] said that the subiculum was smooth, but we find it to be ornamented with small spines (Figure 3(4d)). He also mentioned that the hair walls are smooth, but they are roughened through their whole length as our photos show (Figure 3(3a,3c)). In our description we have shown that living asci and ascospores are larger than Korf’s measurements indicate. This is clearly due to his observation being made on dead material, either because the fresh specimen was mounted in a medium that killed the cells (e.g., KOH) or because observations were made on a specimen that had already been dried and was subsequently rehydrated. Our biometry of dead asci and ascospores agrees with his measurements [1] although he found somewhat longer ascospores—up to 22.5 µm in length—while we found ascospores only up to 18.6 µm length. This is more in keeping with the collection reported by Korf [3], which had somewhat shorter ascospores. It is clear from our images of ascospores (Figure 2(7a–7c)) that the ascospores have at their ends long gelatinous appendages. These are mentioned by Korf [1] for other species of *Arachnopeziza* (such as *A. aurelia*), but not for *A. fitzpatrickii*; probably they are only visible when studying the collection in a living state. Our culture shows similar morphology to the one reported by Korf [1]. The germinated ascospores produce slow growing hyaline mycelia with some chlamydospore-like bodies. Some cells are pigmented with yellowish lipid bodies (Figure 4). Korf mentioned colored pigments in his cultures (*A. aurata*, *A. delicatula*, *A. aurelia*), but not the presence of oxalate crystals like the ones we can see in *A. fitzpatrickii* (Figure 4(3a–3c).

When Korf [1] erected *Arachnopeziza fitzpatrickii*, he discussed the possibility of erecting a new genus for this species based on the peculiar fasciculate hairs only present on the margin of the apothecia. Ultimately, he decided against this action because other species tend to form cohering hairs that look like teeth: *A. aurelia*, *A. leonina* and *A. trabinelloides*. In his monograph he split the genus into five unranked groups: (I) *Typicae* for species with small apothecia, hyaline hairs, thin excipulum and spores 1–7-septate; (II) *Aureliae* for species with large apothecia, orange pigmented hairs, well developed excipulum and spores 1–7-septate; (III) *Arachnoscyphae* for species with delicate hairs, ascospores minute and 1-septate; (IV). *Arachnasterae* for species with hairs cemented into long teeth; and (V) *Anomalae* for species with hairs faintly roughened their whole length. None of these groups are phylogenetically supported in the analyses conducted by Kosonen et al. [5] or our own analyses (Figure 2). Only *A. fitzpatrickii* was included in *Arachnasterae*; the other species that have fasciculate hairs were placed in *Aureliae* (*A. aurelia*, *A. leonina*) or in *Anomalae* (*A. trabinelloides*). The most recently described species with fasciculate hairs was *A. ptilidiophila*, described by Kosonen et al. [5]. *Arachnopeziza fitzpatrickii* is differentiated from these other four species of *Arachnopeziza* by its white apothecia and the biometry of its asci and ascospores (Table 2). Macroscopically, *A. ptilidiophila* is the most similar to *A. fitzpatrickii*; both species have small apothecia, less than 0.7 mm diam, and are whitish. The other three species with teeth have colored hairs on their receptacles. The biometry of the asci in *A. fitzpatrickii* and *A. trabinelloides* overlaps (Table 2), but the ascospores of the former tend to be longer and 3-septate, while the latter has only 1-septate ascospores. *Arachnopeziza aurelia* and *A. leonina* have 3-septate ascospores similar to *A. fitzpatrickii*, but the asci in the former two are larger than *A. fitzpatrickii*. These species are easily differentiated by the size and color of their apothecia (Table 2). The fasciculate hairs in *A. fitzpatrickii* are strictly restricted to the margin in contrast in the other species where they can be formed on the receptacle [1]. Furthermore, only *A. fitzpatrickii*, *A. aurelia* and *A. leonina* have hairs than can reach more than 200 µm, while *A. ptilidiophila* and *A. trabinelloides* never have hairs longer than 130 µm (Table 2). In our analyses, none of the species with fasciculate hairs are phylogenetically related (Figure 2); this agrees with the phylogenetic results in Kosonen et al. [5]. That all of these species are found in different clades suggests that this feature has evolved multiple times within the genus and is not taxonomically informative beyond its use in species differentiation.

*Arachnopeziza fitzpatrickii* was resolved in our analyses as a member of *Arachnopeziza* sensu stricto (Figure 1), but not as a close relative of any sequenced species (Figure 2). More taxon sampling (particularly for extra-Eurasian species) and more sequenced genes are needed to further clarify the phylogenetic relationships among species in *Arachnopeziza*. It is evident from the number of sequenced but undescribed and described but unsequenced taxa that there are unresolved questions around this genus.

An interesting result of our analyses apart from the placement of *A. fitzpatrickii* involves the boundaries of *Arachnopeziza* and Arachnopezizaceae. In the course of selecting sequences for our analyses, it was discovered that sequences of a number of taxa were similar to *Arachnopeziza* or *Eriopezia*. To test their relationships to *Arachnopeziza* and *Eriopezia*, they were included in our first phylogenetic analysis, as were sequences from the sister family to Arachnopezizaceae, Erysiphaceae [19] and a more distant family, Lachnaceae. When included in our analyses (Figure 1), a species of *Clathrosphaerina*, a species of *Dwayaangam* and four species of *Durella* were resolved in Arachnopezizaceae with high support. *Clathrosphaerina zalewskii* is the type species of the genus *Clathrosphaerina*, and so this genus should perhaps be considered to belong to Arachnopezizaceae; unfortunately the LSU sequence used in our analysis was not generated from the holotype (CBS 162.49), for which only an ITS sequence is available for comparison. For *Dwayaangam colodena*, sequences derived from the holotype (CBS 118913) were included in our analyses, suggesting that this species belongs in Arachnopezizaceae. The type of the genus *Dwayaangam*, however, has been shown to belong in *Orbiliaceae* [20], so this species requires generic redisposition. The species *Durella atrocyanea*, *D. macrospora*, *D. melanochlora* and *D. suecica* were placed in Arachnopezizaceae, though not in a monophyletic group. *D. macrospora* and one sequence of *D. melanochlora* were resolved as sister to *Eriopezia caesia*, *D. atrocyanea* was resolved as sister to the clade containing *Arachnopeziza sphagniseda*, and the remaining sequences are scattered about the family. This raises interesting questions about the generic disposition of the genus *Durella* (type species *Durella compressa*) as well as *Eriopezia*. Finally, *A. sphagniseda* was excluded from *Arachnopeziza sensu stricto* with high support, though still resolved in Arachnopezizaceae (Figure 1). This species was first placed in Arachnopeziza by Kosonen et al. [5], who combined *Belonium sphagnisedum* Velen. in *Arachnopeziza*. Their analyses consistently resolved this species as basal to *Arachnopeziza*, with the rest of the genus forming a supported monophyletic group. Our phylogenetic analyses shows (Figure 1) that this species is related to *Durella atrocyanea* and it is not within the clade containing the type species of *Arachnopeziza*. Therefore, we leave this species as *Arachnopeziza sensu lato*. *Arachnopeziza sphagniseda* lacks hairs, the only species with this feature in the genus. This exclusion was also recently shown in Karakehian et al.’s analyses [21]. These authors also showed that the genus *Phragmiticola* belongs in Arachnopezizaceae. New collections and better taxon sampling will be needed to clarify where the species and genera mentioned in this paragraph really belong and to further elucidate their relationships to each other.

## Figures and Tables

**Table 1:** Species used in this study with voucher information and GenBank accession numbers. New sequence indicated in bold. Vouchers from which sequences were used for Figure 1 are indicated by a double dagger (‡), and for Figure 2 by a number sign (#).

Taxon	Voucher	Host	Locality	ITS	LSU
*Albotricha acutipila*	TNS-F-16740 ^‡^	Bamboo culm	Japan	AB481234	LC438571
*Arachnopeziza araneosa*	PDD59980 ^‡#^	*Nothofagus* sp.	New Zealand	MH578553	N/A
*A. araneosa*	PDD74085 ^#^	*Nothofagus* sp.	New Zealand	MH578557	N/A
*A. araneosa*	PDD55510 ^#^	Wood	New Zealand	MH578554	N/A
*A. aurata*	TUR179456 ^‡#^	Hardwood (? *Quercus*)	Denmark	MT231649	MT231649
*A. aurata*	CBS116.54 ^#^	?	France	N/A	MH878241
*A. aurata*	HB 6921b ^#^	*Carpinus betulus*	Luxembourg	OM808919	N/A
*A. aurelia*	KUS-F51520 ^‡#^	*Quercus acutissima*	South Korea	JN033409	JN086712
*A. aurelia*	CBS 117.54 ^#^	?	France	MH857261	MH868796
*A. aurelia*	TNS-F-11211 ^#^	Hardwood	Japan	JN033435	AB546937
*A. aurelia*	CBS127675 ^#^	Hardwood	Japan	MH864618	MH876056
*A. aurelia*	MICH 340548 ^#^	*Quercus* sp. shell	USA, MA	MT913603	N/A
*A. aurelia*	K(M)154087 ^#^	?	UK	MZ159370	N/A
*A. aurelia*	OTU3639 ^#^	Env. sample, soil	Japan	MT596075	N/A
*A. aurelia*	TNS-F-40009 ^#^	*Fagus japonica* leaf	Japan	LC425050	LC429386
*A.* aff. *delicatula*	HB 6961/JPP 21077 ^#^	*Ulex europaeus*	France	OM456213	OM456214
*A.* aff. *delicatula*	5426_620 ^#^	*Pinus sibirica*	Russia	OM614866	N/A
*A. delicatula*	TNS-F-12770 ^#^	Wood	Japan	JN033433	JN086736
*A. delicatula*	TK7076 (S, TUR) ^‡#^	*Populus tremula*	Finland	MT231650	MT231650
*A. delicatula*	JK14051801 (S, TUR) ^#^	*Acer* sp.	USA, MA	MT231651	MT231651
*A. delicatula*	JP6655 (S, TUR) ^#^	*Populus tremula*	Finland	MT231652	MT231652
*A. delicatula*	TK7036 (S, TUR) ^#^	*Betula* sp./*Acer* sp.	Finland	MT231653	MT231653
*A. delicatula*	TNS-F-11212 ^#^	Hardwood	Japan	JN033436	AB546936
*A. delicatula*	TK7115 (S, TUR) ^#^	*Betula* sp.	Finland	MT231654	MT231654
*A. delicatula*	TK7127 (S, TUR) ^#^	*Betula* sp.	Sweden	MT231655	MT231655
*A. delicatula*	TK7152 (S, TUR) ^#^	*Betula* sp.	Sweden	MT231656	MT231656
*A. delicatula*	TENN 074424 ^#^	Wood	USA, SC	MH558278	N/A
*A. delicatula*	Isolate CL0301_4_1 ^#^	Env. sample *Erica* roots	South Africa	KY228700	N/A
*A. delicatula*	JHH 2210 (NYS) ^#^	?	USA, MA	U57496	N/A
*A. delicatula*	CBS127674 ^#^	Hardwood	Japan	MH864617	MH876055
*A. delicatula*	KUS-F52038 ^#^	Hardwood/Softwood	South Korea	JN033393	JN086696
*A. delicatula*	TNS-F-83287/NBRC 115014 ^#^	Hardwood/Softwood	Japan	LC619224	N/A
*A. delicatula*	GBS1 ^#^	?	? China	ON025242	N/A
*A. estonica*	SH15/38 (S, TUR) ^‡#^	*Sphagnum squarrosum*	Estonia	MT231657	MT231657
*A. estonica*	TL210 (TUR) ^#^	*Sphagnum* sp.	Finland	MT231658	MT231658
*A. estonica*	TL245 (TUR) ^#^	*Sphagnum squarrosum*	Finland	MT231659	MT231659
*A. estonica*	TL253 (TUR) ^#^	*Sphagnum* sp.	Finland	MT231660	MT231660
***A. fitzpatrickii***	**LQH-103 (FH)** ^‡#^	***Quercus*** **sp.**	**USA, MA**	**OP353633**	**OP353632**
*A. japonica*	SH06/03 (TUR) ^‡#^	*Sphagnum* sp.	Sweden	MT231661	MT231661
*A. japonica*	RI194 (TUR) ^#^	*Sphagnum* sp.	Finland	MT231662	MT231662
*A. japonica*	RI239 (TUR) ^#^	*Sphagnum* sp.	Finland	MT231663	MT231663
*A. japonica*	TL267 (TUR) ^#^	*Sphagnum* sp.	Finland	MT231664	MT231664
*A. leonina*	KH.15.23 (S, TUR) ^‡#^	*Populus* sp.	Estonia	MT231665	MT231665
*A. leonina*	TK7101 (S, TUR) ^#^	*Populus tremula*	Estonia	MT231666	MT231666
*A. leonina*	SH16/42 (S, TUR) ^#^	*Populus tremula*	Sweden	MT231667	MT231667
*A. leonina*	JYV 11610 ^#^	?	Finland	MT277004	N/A
*A. obtusipila*	TNS-F-12768 ^#^	*Quercus crispula*	Japan	JN033445	JN086746/LC429372
*A. obtusipila*	TNS-F-12769 ^‡#^	*Betula ermanii*	Japan	JN033446	JN086747
*A. ptilidiophila*	TK7287 (S, TUR) ^‡#^	*Ptilidium sp.* on *Pinus*	Finland	MT231668	MT231668
*A. ptilidiophila*	TK7289 (S, TUR) ^#^	*Ptilidium sp.* on *Pinus*	Finland	MT231669	MT231669
*A. ptilidiophila*	TK7290 (S, TUR) ^#^	*Ptilidium sp.* on *Pinus*	Finland	MT231670	MT231670
*A. ptilidiophila*	AL51 (TUR) ^#^	*Ptilidium pulcherrimum*	Finland	MT231671	MT231671
*A. sphagniseda*	RI226 (TUR) ^‡^	*Sphagnum* sp.	Finland	MT231675	MT231675
*A. trabinelloides*	GJO 0071771 ^‡#^	Hardwood	Austria	MT231679	MT231679
*A. trabinelloides*	JK14030203 (S, TUR) ^#^	Hardwood	USA, MA	MT252825	N/A
*Arachnopeziza* sp.	PDD 112238 ^#^	*Dianella nigra*	New Zealand	MH578549	N/A
*Arachnopeziza* sp.	OTU3623 ^#^	Env. sample, soil	Japan	MT596068	N/A
*Arachnopeziza* sp. “a”	RI199 (TUR) ^#^	*Sphagnum* sp.	Finland	MT435517	MT435517
*Arachnopeziza* sp. “a”	TL260 (TUR) ^#^	*Sphagnum* sp.	Finland	MT231672	MT231672
*Arachnopeziza* sp. “a”	OTU19 ^#^	Env. sample, *Picea* forest	Sweden	EF521221	N/A
*Arachnopeziza* sp. “a”	SG031 E06 ^#^	Env. sample, conifer forest	CA, BC	KP889888	N/A
*Arachnopeziza* sp. “a”	OTU18 ^#^	Env. sample, *Picea* forest	Sweden	EF521220	N/A
*Arachnopeziza* sp. “b”	TK7223 (S, TUR) ^#^	*Picea abies*	Sweden	MT231673	MT231673
*Arachnopeziza* sp. “b”	TK7286 (S, TUR) ^#^	*Picea abies*	Finland	MT231674	MT231674
*Arachnopeziza* sp. (1)	JCM 31955 ^#^	Env. sample, root *Castanopsis*	Japan	LC190976	N/A
*Arachnopeziza* sp. (1)	OTU3632 ^#^	Env. sample, soil	Japan	MT596072	N/A
*Arachnopeziza* sp. (2)	c19 ^#^	Env. sample, ericoid root	USA, NC	HM030576	N/A
*Arachnopeziza* sp. (3)	PDD 11223 ^#^	*Phormium* sp. dead leaf	New Zealand	MH578548	N/A
*Arachnopeziza* sp. (4)	EXP-0411F ^#^	Env. sample, *Elaeocarpus* leaf	New Zealand	DQ914728	N/A
*Arachnopeziza* sp. (5)	Strain Mh859.5.8 ^#^	Env. sample, *Diospyros* leaf	USA, NC	GQ996153	N/A
*Arachnopeziza* sp. (6)	PDD 112226 ^#^	*Juncus* sp. leaf	New Zealand	MH578523	N/A
*Arachnopeziza* sp. (7)	PDD 111524 ^#^	*Nothofagus moorei*	Australia	MH578552	N/A
*Arachnopeziza* sp. (8)	PDD 105290 ^#^	Wood	New Zealand	MH578551	N/A
*Arachnopeziza* sp. (9)	PDD 105289 ^#^	*Leptospermum scoparium*	New Zealand	MH578550	N/A
*Blumeria graminis*	MUMH 1707 ^‡^	*Triticum aestivum*	Switzerland	AB273542	AB273542
*Capitotricha attenuans*	E.R.D. 7931 ^‡^	*Rumex suffruticosus*	Spain	MT370339	MT370355
*Clathrosphaerina zalewskii*	CBS 160.51 ^‡^	*Salix* sp. leaf	Netherlands	N/A	MH868311
*Durella atrocyanea*	G.M. 2022-06-28.8 ^‡^	*Cytisus scoparius*	France	OP364398	OP364398
*Du. atrocyanea*	H.B. 7074 ^‡^	*Malus* sp.	Germany	OM456235	N/A
*Du. macrospora*	G.M. 2015-04-05-4 ^‡^	*Crataegus* sp.	Luxembourg	KY462813	KY462813
*Du. macrospora*	H.B. 9974 ^‡^	*Salix* sp.	Germany	KY462812	KY462812
*Du. melanochlora*	Bio-material iNAT:20438540 ^‡^	Wood	US, NY	MN752427	N/A
*Du. melanochlora*	H.B. 6922e ^‡^	*Fagus sylvatica*	Luxembourg	OM456236	N/A
*Du. suecica*	KR-M-053723 ^‡^	?	Germany	MK795727	N/A
*Dwayaangam colodena*	CBS 118912 ^‡^	*Picea mariana* needles	CA, QC	AY746350/MH863042	MH874595
*Dw. colodena*	CBS 118913 ^‡^	*Picea mariana* needles	CA, QC	AY746351/MH863043	MH874596
*Eriopezia caesia*	TK7005 (S, TUR) ^‡#^	?	Finland	MT231685	MT231685
*E. caesia*	G.M. 2015-07-06.2 (LUX) ^‡^	*Quercus* sp.	Luxembourg	MZ322849	MZ322849
*Erioscyphella sinensis*	TNS-F-80354 ^‡^	*Castanopsis sieboldii*	Japan	LC669471	LC533143
*Hymenoscyphus* sp. “b”	TUR 174911 ^‡^	Bryophyte	Finland	EU940222	EU940146
*Incrucipulum foliicola*	TNS-F-81508 ^‡^	*Myrica gale* var. *tomentosa*	Japan	LC438567	LC438584
*Lachnellula resinaria*	G.M. 2015-08-02.1 ^‡^	*Pinus sylvestris*	France	MN719894	MN719894
*Lachnum virgineum*	TNS-F-16583 ^‡^	Wood	Japan	AB481268	AB926119
*Mimicoscypha* aff. *mimica* sp. “a”	CBS 126294/TUR 185321	Bryophyte	Finland	MH863964/EU940197	MH875421/EU940121
*M.* aff. *mimica* sp. “a”	CBS 126293/TUR 185322	Bryophyte	Finland	MH863963/EU940213	EU940137
*Neoerysiphe galeopsidis*	HAL 1206 F ^‡^	*Galeopsis bifida*	Germany	ON415297	ON415297
*Parauncinula polyspora*	MUMH 5223 ^‡^	*Quercus serrata*	Japan	AB985513/LC222319	LC222319
*Pa. septata*	MUMH 4435 ^‡^	*Quercus serrata*	Japan	AB985516/LC222316	LC222316
*Pa. uncinata*	MUMH 4131 ^‡^	*Quercus variabilis*	Japan	AB985510/LC222314	LC222314
*Phyllactinia greyiae*	PRU 130666 ^‡^	*Greyia sutherlandii*	South Africa	OP549039	OP549709
*Proliferodiscus pulveraceus*	G.M. 2017-03-21.3 ^‡^	*Salix cinerea*	Luxembourg	MN066320	MN066320
*Salmonomyces acalyphae*	BRIP 68804 ^‡^	*Acalypha wilkesiana*	Australia	MT133544	MT133550
*Sawadaea bicornis*	WTU-F-0072491 ^‡^	*Acer campestre*	Germany	MT462312	MT462312

**Table 2:** Morphological and biometric comparison among species of *Arachnopeziza* that have fascicles of hairs in their receptacle and/or margin of the apothecia.

Taxon	Apo.	Hairs	Asci	Ascospores	Reference
*Arachnopeziza fitzpatrickii*	0.3–0.6 mm, white	† 200(350) × 2–2.7 µm, fascicles of 10 cemented hairs forming long narrow teeth, hyaline, thick-walled, smooth or with external particles	† 63–75 × 6.8–8.9 µm, 8-spored, amyloid, with croziers	† 13.6–22.5 × 2.7–4.8 µm, hyaline, subfusoid, attenuate below, (1) 3-septate	[1]
*A. fitzpatrickii*		often fewer fascicles, some of 10 cemented hairs		† 12.4–15.4 (21.2) × 2.9–3.3 (4.0) µm	[3]
*A. aurelia*	0.5–3 (5) mm, bright orange	† 100–500 × 1.5–3 µm, often cohering to form short teeth, hyaline to orange walls and contents, smooth, orange pigment dissolving in KOH	† 65–87 × 7.5–8.9 µm, 8-spored, amyloid, with croziers	† 12.2–16.4 (18.4) × 2.7–4.1 µm, hyaline, fusoid-cylindric, attenuated in extremes, 3-septate	[1]
*A. leonina*	0.5–3.5 mm, orange	† 50–150 (250) × 2–4.1 µm, sometimes cohering to form short teeth, tending to branch occasionally, hyaline to yellow-orange walls and contents, smooth, orange pigment dissolving in KOH	† 70–85 × 6.8–8.9 µm, 8-spored, amyloid, with croziers	† (8.9) 10.9–17.1 (19.1) × (2) 2.7–3.4 (4.1) µm, hyaline, subfusiform, attenuate at extremes, (0) 1 (3)-septate	[1]
*A. ptilidiophila*	0.2–0.7 mm, white-yellow	† 80–130 × 3–5 µm, attachedto each other giving a shaggy appearance, hyaline, resin present as small hyaline droplets on the wall.	† 70–100 × 6–12 µm, 8-spored, amyloid, with croziers	† 15.7–23.4 (25.6) × 3.9–5.3 (5.5) µm, inequilateral, 0 (1)-septate	[5]
*A. trabinelloides*	0.3–1.2 mm, whitish to dilute orange	† 55–85 (125) × up to 2 µm, flexuous, sometimes a bit fasciculate, hyaline-yellowish, faintly roughened their whole length with granules	† 60–78 × 6.8–7.5 µm, 8-spored, amyloid, with croziers	† (8.2) 10.9–13.6 (16.4) × (2) 2.7–3.4 (4.8) µm, hyaline, fusoid-elliptical to subfusiform, attenuated below, 1-septate	[1]

## Data Availability

The DNA sequence data presented in this study are available in GenBank under accession numbers OP353633 and OP353632. The examined specimen is deposited in the Harvard University Herbaria (FH) under voucher LQH-103.
